# ALCOdb: Gene Coexpression Database for Microalgae

**DOI:** 10.1093/pcp/pcv190

**Published:** 2015-12-07

**Authors:** Yuichi Aoki, Yasunobu Okamura, Hiroyuki Ohta, Kengo Kinoshita, Takeshi Obayashi

**Affiliations:** ^1^Graduate School of Information Sciences, Tohoku University, 6-3-09, Aramaki-Aza-Aoba, Aoba-ku, Sendai, 980-8579 Japan; ^2^Core Research for Evolutional Science and Technology (CREST), Japan Science and Technology Agency (JST), Kawaguchi, Saitama, Japan; ^3^Department of Biological Sciences, Tokyo Institute of Technology, Yokohama, Kanagawa, 226-8501 Japan; ^4^Earth-Life Science Institute, Tokyo Institute of Technology, 2-12-1 Ookayama, Meguro-ku, Tokyo, 152-8551 Japan; ^5^Institute of Development, Aging, and Cancer, Tohoku University, Sendai, 980-8575 Japan; ^6^Tohoku Medical Megabank Organization, Tohoku University, Sendai, 980-8573 Japan

**Keywords:** Comparative transcriptomics, *Chlamydomonas reinhardtii*, *Cyanidioschyzon merolae*, Database, Gene coexpression, Microalgae

## Abstract

In the era of energy and food shortage, microalgae have gained much attention as promising sources of biofuels and food ingredients. However, only a small fraction of microalgal genes have been functionally characterized. Here, we have developed the Algae Gene Coexpression database (ALCOdb; http://alcodb.jp), which provides gene coexpression information to survey gene modules for a function of interest. ALCOdb currently supports two model algae: the green alga *Chlamydomonas reinhardtii* and the red alga *Cyanidioschyzon merolae.* Users can retrieve coexpression information for genes of interest through three unique data pages: (i) Coexpressed Gene List; (ii) Gene Information; and (iii) Coexpressed Gene Network. In addition to the basal coexpression information, ALCOdb also provides several advanced functionalities such as an expression profile viewer and a differentially expressed gene search tool. Using these user interfaces, we demonstrated that our gene coexpression data have the potential to detect functionally related genes and are useful in extrapolating the biological roles of uncharacterized genes. ALCOdb will facilitate molecular and biochemical studies of microalgal biological phenomena, such as lipid metabolism and organelle development, and promote the evolutionary understanding of plant cellular systems.

## Introduction

Microalgal species have attracted worldwide attention as potentially important sources of biofuel feedstock (Wijffels and Barbosa 2010), animal feed and even human nutrients (Draaisma et al. 2013). Particularly in recent years, microalgae-based renewable fuels have been actively studied (Gimpel et al. 2013) because of global concerns regarding a sustainable energy supply. In such studies, the identification of genes involved in oil synthesis and turnover is an important step in achieving increased biofuel production from microalgae. In addition to their industrial impact, microalgae are also important organisms in the investigation of the evolution of plant cellular processes, such as plastid biogenesis (Reyes-Prieto et al. 2007) and cell wall development (Popper et al. 2011). Despite these important aspects, only a small fraction of microalgal genes have been functionally characterized (Supplementary Fig. S1), and thus bioinformatics support is needed to determine the functions of the remaining genes.

Gene coexpression analysis is a powerful approach to infer the functions of uncharacterized genes based on the similarity of gene expression profiles. This approach is based on the guilt-by-association principle in that it assumes that functionally related genes are generally expressed co-ordinately in spatial–temporal states or across different environmental conditions. It has been successful in clarifying various biological phenomena, especially in the plant sciences (Usadel et al. 2009, Hansen et al. 2014). Currently, there are several gene coexpression databases focusing on a variety of model species, including human, mouse, Arabidopsis and rice (Obayashi et al. 2014, Ohyanagi et al. 2014, van Dam et al. 2014, Okamura et al. 2015). With attractive service and user interfaces that (i) provide maintained and enhanced data through regular updates; (ii) evaluate data reliability; and (iii) enable advanced data interpretations, such as interspecies comparisons and network analyses, these databases have facilitated the functional characterization of plant and animal genes. For microalgae, in contrast, there has been no gene coexpression database that fulfills these functional requirements. Although the AlgaePath database (Zheng et al. 2014) provides gene coexpression information about some green algae, it specializes in expression profile analyses of individual genes, and whole coexpression information is not available.

To overcome this deficiency, we decided to construct a comprehensive gene coexpression database for microalgal species. Thus, we prepared gene coexpression data for two model algae, *Chlamydomonas reinhardtii* and *Cyanidioschyzon merolae. C. reinhardtii* is a unicellular green alga, which has been widely used as a model species for studying photosynthesis (Merchant et al. 2007) and as a test species for biofuel production (Scranton et al. 2015). The red unicellular alga *C. merolae* is a model organism for investigating the regulatory mechanisms of organelle division (Matsuzaki et al. 2004). Here, we describe the development of a new database named the Algae Gene Coexpression database (ALCOdb; http://alcodb.jp), which provides microalgal gene coexpression information with a user-friendly interface. The database contains several unique characteristics for enabling interspecies comparisons, network analyses and a combined approach with differential expression analyses. ALCOdb will facilitate microalgal research at the molecular level and promote the evolutionary understanding of cellular systems from microalgae to higher plants.

## Results and Discussion

### Preparation of microalgae gene coexpression data

For construction of the coexpression database for microalgae, we selected two species, the green alga *C. reinhardtii* and the red alga *C. merolae*, based on the availability of gene expression data. We downloaded >300 public RNA sequencing (RNA-seq) data sets for *C. reinhardtii* from the Sequence Read Archive in the DNA Data Bank of Japan (Kodama et al. 2012). For *C. merolae*, we obtained two large microarray-based gene expression data sets from the Gene Expression Omnibus (Barrett et al. 2013). The collected data were converted to gene expression matrices (for details, see the Materials and Methods), and then gene coexpression for each species was calculated. As the platforms and biological phenomena focused on were very different, we separately calculated gene coexpression data for the two *C. merolae* experimental platforms. We used Mutual Rank (MR) to quantify the similarities of gene expression profiles because MR performs better in the Gene Ontology (GO) annotation prediction than the raw Pearson’s correlation coefficient (PCC) value (Obayashi and Kinoshita 2009). The details of the coexpression data are summarized in Table 1.

**Table 1:** A summary of gene coexpression data in ALCOdb

Species	Version	No. of genes	Gene model	No. of samples*^a^*	GO score*^b^*	Codon score*^c^*	Data source	Release date
*C. reinhardtii*	Cre-R1‐15‐08	15,519	JGI v5.5	172	6.07	1.68	RNA-seq	August 24, 2015
	Cre-R1‐13‐10	18,773	JGI v5.5	137	5.17	1.48	RNA-seq	
*C. merolae*	Cme-M1‐14‐06	4,586	*C. merolae* Genome Project	75	0.90	0.00	Micrroarray (GSE8268)	September 17, 2014
	Cme-M2‐14‐06	6,506	*C. merolae* Genome Project	48	1.35	1.31	Micrroarray (GSE37673)	September 17, 2014

*^a^* The number of slides for each microarray experiment or the number of runs per RNA-seq experiment.

*^b^* The predictive performance of the GO annotation as represented by the AUROC_0.01_ (E-04). A larger score indicates a better performance.

*^c^* The coincidence score with codon similarity as represented by the median of the COXSIM value (E-02). A larger score indicates a better performance.

Because the efficacy of a gene coexpression analysis in microalgal studies is unclear, even in model species such as *C. reinhardtii*, it is necessary to understand how gene coexpression can detect functionally related genes. In addition, the sample coverage of gene expression data might affect the predictive power directly, and thus the quality assessment of coexpression data is also important. Therefore, we conducted a performance evaluation of the coexpression data (for details, see the Materials and Methods).

For this purpose, we calculated the degree of association between gene coexpression and gene function similarity, which was previously designed as the GO score (Obayashi et al. 2014) (Table 1). The results indicate that all of the coexpression data can identify functionally related genes to some extent, because the GO score is theoretically equal to 0.50E-04 if there is no potential to discriminate gene pairs correctly (McClish 1989). For a comparison, we also tested the initial version of the *C. reinhardtii* coexpression data (Cre-R1‐13‐10), which was calculated from a smaller amount of RNA-seq data. The score using the latest version of the coexpression data (Cre-R1‐15‐08) was superior to that of the initial version (Table 1). This indicates that the GO scores reflected the improved performance that resulted from better sample coverage of the gene expression data. For the *C. merolae* data sets, Cme-M2‐14‐06 performed better than Cme-M1‐14‐06 (Table 1). This might reflect the differences in the biological phenomena focused on in each microarray experiment. Cme-M1‐14‐06 was calculated from the microarray data that focused on the cell cycle, whereas Cme-M2‐14‐06 was prepared from data measuring the gene expression dynamics in response to environmental changes. The latter might have captured the gene coexpression with higher resolution compared with the former, possibly because of fluctuations in the majority of genes. Given this result, we recommend the use of Cme-M2‐14‐06 data for *C. merolae* gene coexpression analyses at this stage. The GO annotations used in this analysis were retrieved from the Phytozome database (Goodstein et al. 2012) and the *C. merolae* genome project website (Matsuzaki et al. 2004).

Although calculating GO scores is a straightforward approach for evaluating the overall performance of coexpression data, it does not take into account poorly annotated genes, i.e. genes that rarely have validated GO terms. To overcome this limitation, we calculated the coincidence score of codon similarity, termed the ‘Codon score’ in a previous study (Obayashi et al. 2014), for every gene in each coexpression data set. As shown in Table 1, the representative values (the median of the Codon scores for each data set) showed the same patterns as the GO scores, and thus we confirmed the results described above. Furthermore, as the Codon score was highly correlated with the GO score, it suggested that the similarity in codon usage is a strong indicator of gene coexpression in microalgae. This will be helpful in assessing coexpression data for non-model algae with GO annotations that might be quite poor. Note that the Codon scores for the microalgae are in the same range as those for the higher plants provided in ATTED-II ver 8.0 (http://atted.jp/top_statistics.shtml).

We will continue to improve our data using these scores as quality indices.

### Fundamental functions of ALCOdb

**Fig. 1:**
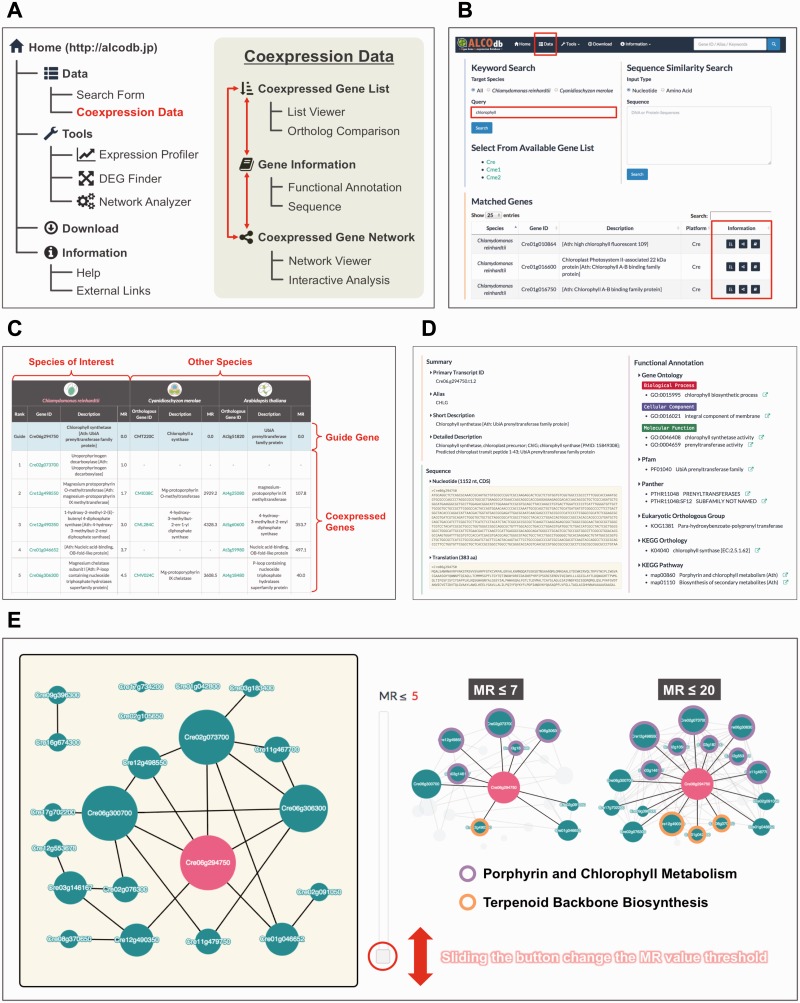
An overview and the fundamental functions of ALCOdb. (A) The site diagram for ALCOdb. (B) The search form and an example of the search results when querying the keyword ‘chlorophyll’. The coexpression data can be accessed by clicking the link icons displayed in the right part of the search results. (C–E) An example of coexpression information pages with some modifications. *Cre06g294750* is used as an example of a guide gene. For a guide gene, ALCOdb provides three unique pages: a ‘Coexpressed Gene List’ page (C), a ‘Gene Information’ page (D) and a ‘Coexpressed Gene Network’ page (E). Red text and boxes are used here to highlight certain aspects of this application.

To present our coexpression data in an easily understood manner, we developed a web-based database named ALCOdb (http://alcodb.jp). The site diagram of ALCOdb is shown in Fig. 1A. In summary, ALCOdb provides three unique information pages for each gene: (i) Coexpressed Gene List; (ii) Gene Information; and (iii) Coexpressed Gene Network. Users can navigate back and forth among these pages smoothly and acquire an insight into the biological roles of their genes of interest. These pages can be accessed from the ‘Data’ menu (located in the top menu bar) through a search form (Fig. 1B). Users can search the genes of interest by any keywords or sequence similarity. If there are any hits, then the links to information pages are provided. Alternatively, users can select a target from the available gene list. Details and examples about each information page are described below.

The ‘Coexpressed Gene List’ page provides coexpressed genes in a list, along with their orthologous relationships (Fig. 1C). Four columns on the left show the coexpressed genes, which are sorted in ascending order of their MR values, in the species of interest (*C. reinhardtii*, in this case). A smaller MR value indicates a stronger degree of coexpression. Users can deduce the function of the guide gene based on the functional annotations of higher ranked genes. The six columns on the right indicate the degrees of coexpression for orthologous gene pairs in other species (*Arabidopsis thaliana* and *C. merolae*, in this case). These columns are available only when the guide gene has orthologous genes in the corresponding species. We implemented this function to provide a comparative view of the coexpressed genes. We also incorporated the coexpression data of *A. thaliana*, which were provided by the ATTED-II database (Obayashi et al. 2014), to allow an interspecies comparison among microalgae and higher plants. Orthologous genes were defined by reciprocal best BLASTP hits (Tatusov et al. 1997).

The ‘Gene Information’ page provides a functional annotation and sequence for each guide gene (Fig. 1D). The functional annotation consists of the following classification terms: Gene Ontology (Blake et al. 2013), Pfam (Finn et al. 2014), Panther (Mi et al. 2013), Eukaryotic Orthologous Group (Tatusov et al. 2003), KEGG Orthology and KEGG Pathway (Kanehisa et al. 2012). All annotations provided on this page were retrieved from the Phytozome database and the *C. merolae* genome project website. Each sequence includes the coding DNA sequence and its deduced protein. For *C. reinhardtii* genes, information on their orthologous genes in *A. thaliana* was imported to enhance the functional annotation coverage. This effectively increased the number of annotated genes (22–50%, see Supplementary Fig. S1A) and also made it easier to interpret the ‘Coexpressed Gene List’ page because of the reduction in blank cells. Users can explore more detailed information for each classification term by clicking on the external links icon next to each annotation.

The ‘Coexpressed Gene Network’ page provides another view of coexpressed genes by displaying them in a network with interactive analysis tools (Fig. 1E). Network representations of gene interactions have provided us with unforeseen insights into complex biological functions (Barabási and Oltvai 2004). The red node indicates the guide gene, and green nodes indicate coexpressed genes. The node degree (i.e. the number of adjacent nodes) is reflected in the node size, allowing for the easier discovery of network hub genes. To generate a network, the five most strongly coexpressed genes for a guide gene and the three most strongly coexpressed genes for each of those genes, resulting in 20 genes surrounding a guide gene, are selected. Then, any gene pair whose MR value is not greater than a given threshold is connected. Users can change the threshold interactively on the browser and obtain a resulting network. This strategy enables users to find functional modules (e.g. sets of genes sharing a common KEGG Pathway) and to estimate quantitatively the function of a guide gene. In this case, the coexpression network contains two functional modules: (A) Porphyrin and Chlorophyll Metabolism and (B) Terpenoid Backbone Biosynthesis. By changing the threshold of the MR value, it was revealed that *Cre06g294750* is more functionally related to module A than to module B. This reflects the fact that Cre06g294750 protein is directly involved in chlorophyll (Chl) biosynthesis and that a phytyl diphosphate (a terpenoid compound) was required in this reaction (Schmid et al. 2002). The network sections of this page are implemented by Cytoscape.js (Lopes et al. 2010), and the resultant graph can be saved as a PNG image file.

The raw coexpression data can be downloaded freely from the ‘Download’ page (Fig. 1A). This page also provides details for each platform, as shown in Table 1.

### Advanced functions of ALCOdb

ALCOdb also provides several useful tools that enable users to perform advanced analyses. These tools are available from the ‘Tools’ menu.

**Fig. 2:**
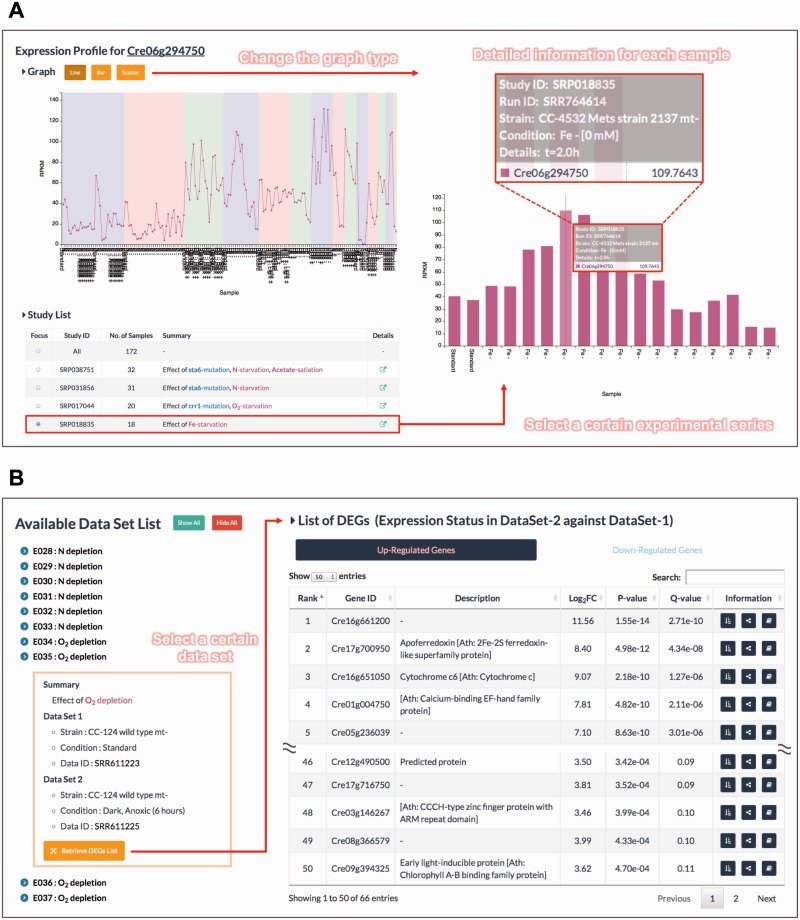
The advanced functions of ALCOdb. (A) An example of the ‘Expression Profiler’ tool. *Cre06g294750* is used as an example query gene. The line graph shows the overall expression profile and is color-coded as in the experimental series (termed Study). The bar graph shows the detailed expression profile from a study focused on the effects of iron deficiency (SRP018835). When the cursor is moved over a sample in the expression profile, its corresponding detailed information is displayed. The expression level was calculated using the reads per kilobase of exon per million reads (RPKM) metric. (B) An example of the ‘DEG Finder’ tool. The DEGs list shows the highly up-regulated genes under dark and anoxic conditions (SRR611225) relative to standard conditions (SRR611223). The list contains not only the *P*-values but also *Q*-values to allow for a flexible definition of DEGs with an arbitrary FDR. In this case, if the top 50 genes in the list are regarded as DEGs (the *Q*-value of the 50th ranking gene is 0.11), then there will be at least five false positives.

The ‘Expression Profiler’ is useful for exploring the detailed expression profile of a gene of interest (Fig. 2A). RNA-seq metadata for *C. reinhardtii* were manually curated and summarized into the following key points: Strain (genetic effects), Condition (environmental effects) and Details (such as time course and concentration of chemicals). When a user’s mouse hovers on the graph, the corresponding sample information is displayed. The expression profile consists of several experimental series, and each series can be interactively examined. With this tool, users can find the conditions when a gene of interest was expressed at high (or low) levels. Because we re-mapped every short read derived from old RNA-seq data against the latest gene models, this tool is valuable in providing the most accurate gene expression levels in a variety of samples.

The ‘DEG Finder’ provides a set of genes that are differentially expressed under certain conditions (Fig. 2B). This tool currently provides a differentially expressed gene (DEG) list of *C. reinhardtii* for >100 conditions. The identification of DEGs is a key step in understanding the molecular mechanisms underlying specific biological processes (Garber et al. 2011). DEGs were detected using the TCC package of R software (Sun et al. 2013), and the most highly up- and down-regulated genes (the top 100 genes with statistical significance) are provided. The resulting list contains the following items: Rank, Gene ID, Description, Fold Change (FC; base-2 logarithm converted), *P*-value, *Q*-value and links to each coexpression information page. The *Q*-value corresponds to the minimum false discovery rate (FDR) calculated when a gene and the preceding genes were regarded as DEGs. This tool allows users a flexible (i.e. they can set an arbitrary FDR threshold) search of DEGs associated with various biological phenomena.

The ‘Network Analyzer’ enables users to draw a coexpression network for the user-defined set of genes. A network is displayed in the same style as the ‘Coexpressed Gene Network’ page. Using this tool, users can explore the functional modules concealed in their focused gene set, such as a DEGs list obtained from the ‘DEG Finder’.

### Usage scenario: extrapolate the function of *Cre06g306300*

As an example of ALCOdb use, consider the functional estimation of causative genes for a certain mutant phenotype. *Chlamydomonas* mutant strain *5A7*, which exhibits a light-sensitive phenotype and reduced Chl content, was isolated by screening photosynthesis- and pigment-deficient mutants (Grovenstein et al. 2013). The authors confirmed that this phenotype is due in part to a defect in *Cre06g306300* expression. In the *5A7* strain, the expression level of this gene was extremely low compared with that in the wild-type (parental) strain. Therefore, it is assumed that *Cre06g306300* is involved in Chl biosynthesis and regulates appropriate photosynthetic activities.

**Fig. 3:**
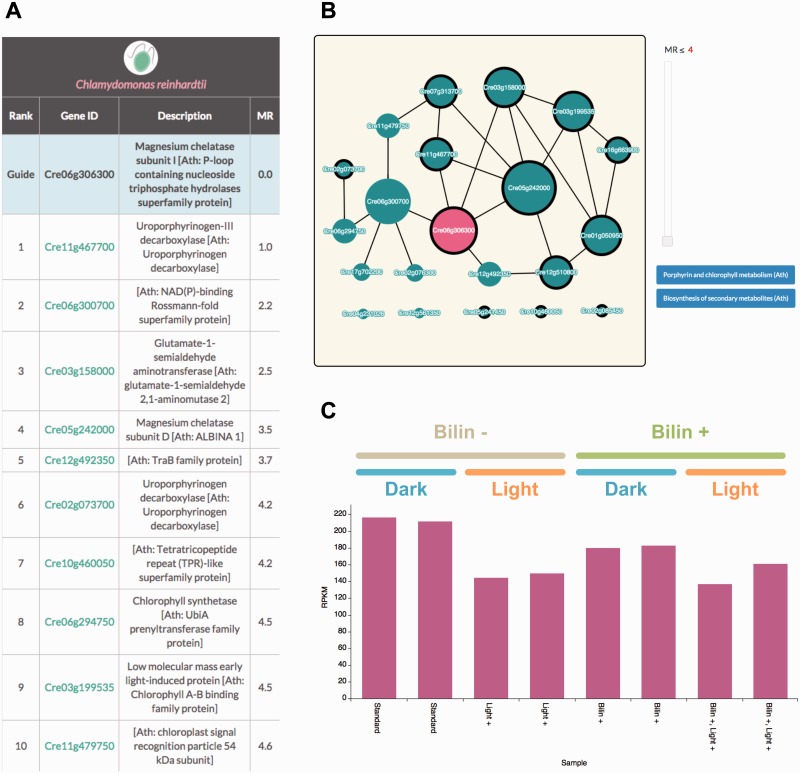
An example using ALCOdb to extrapolate the function of a gene. Coexpression analysis for *Cre06g306300.* (A) The ‘Coexpressed Gene List’ page. Only the top 10 coexpressed genes of *C. reinhardtii* are displayed. The entire list is available at http://alcodb.jp/coexpression/Cre/Cre06g306300/list. (B) The ‘Coexpressed Gene Network’ page. A set of genes annotated as ‘porphyrin and chlorophyll metabolism’ (KEGG map00860) is enclosed by a thick line. This network graph is available at http://alcodb.jp/coexpression/Cre/Cre06g306300/network. (C) The ‘Expression Profiler’ page with some modifications. The study which focused on the effects of the dark to light transition (SRP014795) is displayed. This expression profile is available at http://alcodb.jp/coexpression/ExpressionProfiler?query=Cre06g306300&target=Cre06g306300.

To test this hypothesis, we focused on the set of genes that was highly coexpressed with *Cre06g306300.* When checking the ‘Coexpressed Gene List’ page, we noticed that there were several Chl biosynthesis-related genes in the top part of the list (Fig. 3A). For example, both uroporphyrinogen-III decarboxylase (the first ranked gene) and glutamate-1-semialdehyde aminotransferase (the third ranked gene) encode catabolic enzymes involved in Chl metabolism (Stenbaek and Jensen 2010). Furthermore, the GO terms ‘chlorophyll biosynthetic process’ and ‘porphyrin-containing compound biosynthetic process’ were over-represented in the top 100 coexpressed genes with statistical significance (*P*-values were 2.06E-07 and 2.00E-11, respectively). This feature was also observed on the ‘Coexpressed Gene Network’ page. The coexpression network mainly consists of genes associated with the KEGG Pathway term ‘porphyrin and chlorophyll metabolism’ (Fig. 3B). Additionally, we investigated the expression pattern of *Cre06g306300* using the ‘Expression Profiler’ tool. As shown in Fig. 3C, the expression of *Cre06g306300* was down-regulated during the dark to light transition or with the addition of bilins (linear tetrapyrroles that contribute to the light-harvesting process) (Mochizuki et al. 2010). This result suggests that *Cre06g306300* is a light-responsive gene that affects tetrapyrrole metabolic processes. From these observations, the involvement of *Cre06g306300* in the Chl biosynthetic pathway becomes more likely at the molecular genetic level.

In fact, the Cre06g306300 protein is one of the subunits of magnesium chelatase, which catalyzes a crucial step in the Chl biosynthetic pathway (Ikegami et al. 2007, Stenbaek and Jensen 2010). Focusing again on the coexpressed genes, *Cre06g306300* is tightly coexpressed with *Cre05g242000* (ranked fourth on the list), which is another subunit of Mg-chelatase (Fig. 3A). These results strongly suggest that our coexpression data have the potential to detect functionally related genes correctly.

### Conclusion and future directions

In this study, we prepared gene coexpression data for *C. reinhardtii* and *C. merolae.* To the best of our knowledge, this is the first report on constructing large-scale gene coexpression data for microalgae, especially *C. merolae.* We also established the web database ALCOdb to provide the data and several user-friendly analysis platforms. We will continue to improve the quality of our data by regularly incorporating public gene expression data and improving the calculation methods.

At the present time, ALCOdb supports only two model algae. As mentioned in the Introduction, there is an urgent need to uncover gene functions in industrially important non-model algae. We plan to calculate the gene coexpression of such species based on those of the model algae prepared in this study. We are also developing a new method to estimate the coexpressed gene pairs based on their sequence similarities, which will permit a gene coexpression analysis for any species with available genome sequences.

Due to the relatively simple gene repertoire, microalgal gene coexpression data have the potential to shed light on the function of genes that belong to a large gene family in higher plants. With the above efforts, we will enhance the functionality of ALCOdb to facilitate research on both microalgae and higher plants.

## Materials and Methods

### Construction of gene coexpression data

To generate gene coexpression data for *C. reinhardtii*, we downloaded RNA-seq data from the DDBJ Sequence Read Archive (http://trace.ddbj.nig.ac.jp/dra/). These data were converted to the FASTQ format, and sequence reads were mapped onto the coding sequences of *C. reinhardtii* using Bowtie2 (Langmead and Salzberg 2012). The latest gene models for *C. reinhardtii* (v5.5) were obtained from the Phytozome database (Merchant et al. 2007, Goodstein et al. 2012). Lower quality data (total mapped counts <10,000,000) were filtered out, leaving 172 runs that correspond to 15 experiments. Mapped counts were summed for each gene model and used as the gene expression values. Genes with low levels of expression, i.e. average counts <30, were omitted. After conversion to a base-2 logarithm with a pseudo count of 1, quantile normalization was applied to the data from each experiment using the preprocessCore package with R software, and the average of normalized expression levels was subtracted for each gene. Using all of the experiments at once, PCCs for every gene pair were calculated, and these values were converted to MR values (Obayashi and Kinoshita 2009).

To generate gene coexpression data for *C. merolae*, we downloaded microarray data from the Gene Expression Omnibus (http://www.ncbi.nlm.nih.gov/geo/). There were two large experimental series, GSE8268 (Ujiwara et al. 2009) and GSE37673, consisting of 75 and 48 samples, respectively. The gene expression matrices were obtained after LOWESS normalization and RMA normalization, respectively. For each data set, the MR values of every gene pair were calculated separately.

The latest version of the gene coexpression data for *A. thaliana* (Arabidopsis c5.0) was downloaded from the ATTED-II database (Obayashi et al. 2014).

### Orthology computation and functional annotation

The coding DNA sequences and corresponding protein sequences of *C. reinhardtii* (v5.5) were retrieved from the Phytozome database (Merchant et al. 2007, Goodstein et al. 2012). The *C. merolae* sequences were retrieved from the *C. merolae* genome project website (http://merolae.biol.s.u-tokyo.ac.jp). The sequence set for *A. thaliana* was retrieved from the TAIR database (Lamesch et al. 2012). To identify the orthologous gene pairs, we performed BLASTP searches of all proteome pairs among the three species. Then, orthologous gene pairs were selected based on the reciprocal best hits criterion (Tatusov et al. 1997) using an e-value cut-off of 1E-5. Functional annotation of proteins for each species were also retrieved from the same databases and assigned to individual genes.

### Performance evaluation of gene coexpression data

To evaluate the quality of our coexpression data, the coincidence score between the gene coexpression and the codon usage similarity was calculated, as reported previously (Obayashi et al. 2014). Briefly, a 61 dimension vector was constructed from the codon frequency of each gene, and the similarities of vectors for any gene pair were calculated by MR. Then, for each gene, the other genes were sorted by descending MR values. Finally, the resultant gene list was compared with the corresponding coexpressed gene list using the COXSIM score (Okamura et al. 2015), which is a similarity measure of two ordered gene lists. In the COXSIM calculation, we set parameter *k* to the number of top 1% of genes in each data set. Because the COXSIM value was calculated for every gene, the median of the COXSIM values was adopted as the overall performance of each data set.

### RNA-seq data analysis

The *C. reinhardtii* RNA-seq metadata were obtained from the DNAnexus site (http://sra.dnanexus.com/) and manually curated. Then, the following items were assigned to each sample: strain, condition and details. The full results are shown in Supplementary Table S2. The expression level of every gene in each sample was calculated using the reads per kilobase of exon per million reads (RPKM) expression metric (Mortazavi et al. 2008).

The DEGs were identified using the TCC package (Sun et al. 2013) with R software. When both of the pair of samples had replicates, the following parameters were used: -norm.method tmm -test.method edger -iteration 3 -FDR 0.1 -floorPDEG 0.05 in the ‘calcNormFactors’ command and -test.method edger -FDR 0.1 in the ‘estimateDE’ command. Otherwise, if one or both of the pair of samples had no replicates, the following parameters were used: -norm.method deseq -test.method deseq -iteration 3 -FDR 0.1 -floorPDEG 0.05 in the ‘calcNormFactors’ command and -test.method deseq -FDR 0.1 in the ‘estimateDE’ command. Then, the top 100 genes with the lowest *P*-values were selected to create the list of DEGs. All of the sample pairs used to compute DEGs are shown in Supplementary Table S3.

The *P*-values of over-represented GO terms in the top 100 coexpressed gene set were calculated using the phyper function of R software and adjusted by Bonferroni correction, which is also represented in the coexpressed gene list in ALCOdb.

### Database implementation

ALCOdb was implemented in a Python-based web application framework, Django (https://www.djangoproject.com), with SQLite as the backend database. The ‘Coexpressed Gene Network’ pages and the ‘Network Analyzer’ pages were generated using Cytoscape.js v2.5 (Lopes et al. 2010). ALCOdb was tested to work properly in the following web browsers: Google Chrome 44, Safari 9, Firefox 41 and Internet Explorer 10.

## Supplementary data

Supplementary data are available at PCP online.

## Funding

This research was supported by the Japan Science and Technology Corporation [CREST research project 11102558 to T.O., Grants-in-Aid for Innovative Areas (4114005 to T.O.) and for Scientific Research (15K20863 to Y.A. and 15K18464 to T.O.)].

## Supplementary Material

Supplementary Data

## Abbreviations

ALCOdb: Algae Gene Coexpression Database
Chl: chlorophyll
DEG: differentially expressed gene
FDR: false discovery rate
GO: Gene Ontology
MR: Mutual Rank
PCC: Pearson’s correlation coefficient
RNA-seq: RNA sequencing
RPKM: reads per kilobase of exon per million reads
