# Supplementary material for: ALCOdb: Gene Coexpression Database for Microalgae
Source: Plant Cell Physiol. 2015 Dec 7;57(1):e3. doi: 10.1093/pcp/pcv190 (PMC4722175; doi:10.1093/pcp/pcv190)
Supplement: Supplementary Data [file supp_57_1_e3__index.html]

ALCOdb: Gene Coexpression Database for Microalgae — ALCOdb: Gene Coexpression Database for Microalgae — ALCOdb: Gene Coexpression Database for Microalgae — Supplementary Data 

# ALCOdb: Gene Coexpression Database for Microalgae

## Supplementary Data

files

- Supplementary Data - zip file
