# Supplementary material for: ALCOdb: Gene Coexpression Database for Microalgae
Source: Plant Cell Physiol. 2015 Dec 7;57(1):e3. doi: 10.1093/pcp/pcv190 (PMC4722175; doi:10.1093/pcp/pcv190)
Supplement: Supplementary Data [file supp_pcv190_suppl_data.zip › pcp-2015-e-00476-File005.pdf]

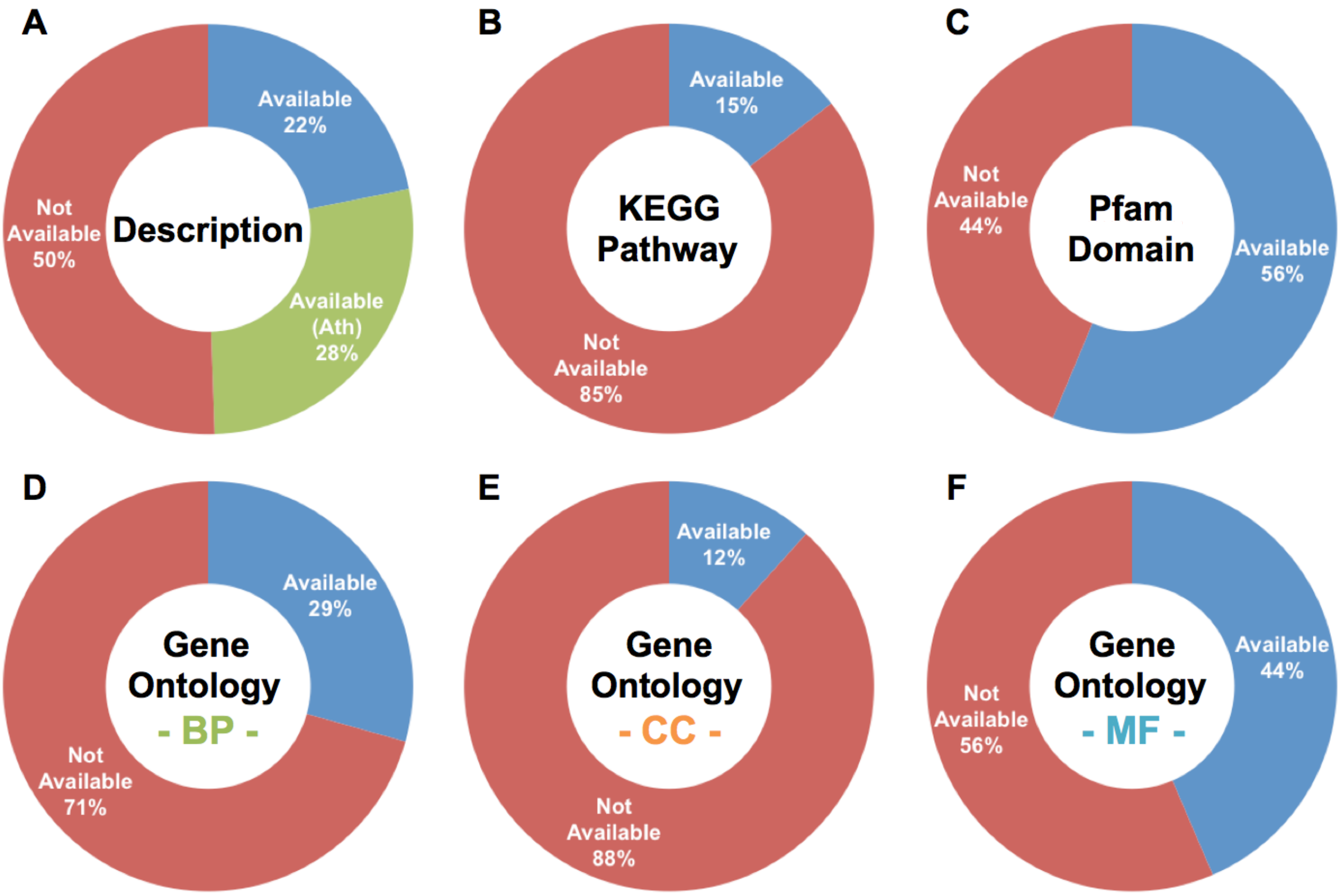

Supplementary Figure S1: The functional annotation coverage of the *C. reinhardtii* transcriptome. The 15,519 genes supported in the Cre-R1-15-08 coexpression data were the subject of this investigation. (A) Descriptions. (B) KEGG Pathway terms. (C) Pfam Domain terms. (D) GO terms in 'Biological Process' (BP). (E) GO terms in 'Cellular Component' (CC). (F) GO terms in 'Molecular Function' (MF). The 'Available' section in each pie chart corresponds to the proportion of genes with annotation terms and 'Not Available' section corresponds to that of genes without any annotation. The 'Ath' section in (A) indicates the proportion of genes that not have any original annotation but assigned some external annotation deriving from the orthologous genes in *A. thaliana*. The functional annotation data were retrieved from the Phytozome database.
