# Supplementary material for: ALCOdb: Gene Coexpression Database for Microalgae
Source: Plant Cell Physiol. 2015 Dec 7;57(1):e3. doi: 10.1093/pcp/pcv190 (PMC4722175; doi:10.1093/pcp/pcv190)
Supplement: Supplementary Data [file supp_pcv190_suppl_data.zip › pcp-2015-e-00476-File006.pdf]

Supplementary Table S2: A summary of manually curated C. reinhardtii RNA-seq metadata

Study ID	Run ID	Sample ID	Study Summary	Strain	Condition	Details
SRP038751	SRR1174384	SRS560402	Starch-null, Acetate-addition (Time Course)	CC-4349 cw15 mt-	Standard	
SRP038751	SRR1174385	SRS560403	Starch-null, Acetate-addition (Time Course)	CC-4349 cw15 mt-	N - [HS -N]	t=0.0h
SRP038751	SRR1174386	SRS560404	Starch-null, Acetate-addition (Time Course)	CC-4349 cw15 mt-	N - [HS -N]	t=0.5h
SRP038751	SRR1174387	SRS560406	Starch-null, Acetate-addition (Time Course)	CC-4349 cw15 mt-	N - [HS -N]	t=2.0h
SRP038751	SRR1174388	SRS560405	Starch-null, Acetate-addition (Time Course)	CC-4349 cw15 mt-	N - [HS -N]	t=4.0h
SRP038751	SRR1174389	SRS560407	Starch-null, Acetate-addition (Time Course)	CC-4349 cw15 mt-	N - [HS -N]	t=8.0h
SRP038751	SRR1174390	SRS560408	Starch-null, Acetate-addition (Time Course)	CC-4349 cw15 mt-	N - [HS -N]	t=12h
SRP038751	SRR1174391	SRS560409	Starch-null, Acetate-addition (Time Course)	CC-4349 cw15 mt-	N - [HS -N]	t=24h
SRP038751	SRR1174392	SRS560410	Starch-null, Acetate-addition (Time Course)	CC-4349 cw15 mt-	N - [HS -N]	t=48h
SRP038751	SRR1174393	SRS560411	Starch-null, Acetate-addition (Time Course)	CC-4349 cw15 mt-	N - [HS -N], Acetate + [20 mM]	t=0.0h
SRP038751	SRR1174394	SRS560412	Starch-null, Acetate-addition (Time Course)	CC-4349 cw15 mt-	N - [HS -N], Acetate + [20 mM]	t=0.5h
SRP038751	SRR1174395	SRS560413	Starch-null, Acetate-addition (Time Course)	CC-4349 cw15 mt-	N - [HS -N], Acetate + [20 mM]	t=2.0h
SRP038751	SRR1174396	SRS560414	Starch-null, Acetate-addition (Time Course)	CC-4349 cw15 mt-	N - [HS -N], Acetate + [20 mM]	t=4.0h
SRP038751	SRR1174397	SRS560415	Starch-null, Acetate-addition (Time Course)	CC-4349 cw15 mt-	N - [HS -N], Acetate + [20 mM]	t=8.0h
SRP038751	SRR1174398	SRS560416	Starch-null, Acetate-addition (Time Course)	CC-4349 cw15 mt-	N - [HS -N], Acetate + [20 mM]	t=12h
SRP038751	SRR1174399	SRS560417	Starch-null, Acetate-addition (Time Course)	CC-4349 cw15 mt-	N - [HS -N], Acetate + [20 mM]	t=24h
SRP038751	SRR1174400	SRS560418	Starch-null, Acetate-addition (Time Course)	CC-4349 cw15 mt-	N - [HS -N], Acetate + [20 mM]	t=48h
SRP038751	SRR1174401	SRS560419	Starch-null, Acetate-addition (Time Course)	CC-4348 sta6-1 mt+	Standard	
SRP038751	SRR1174402	SRS560420	Starch-null, Acetate-addition (Time Course)	CC-4348 sta6-1 mt+	N - [HS -N]	t=0.0h
SRP038751	SRR1174403	SRS560421	Starch-null, Acetate-addition (Time Course)	CC-4348 sta6-1 mt+	N - [HS -N]	t=0.5h
SRP038751	SRR1174405	SRS560423	Starch-null, Acetate-addition (Time Course)	CC-4348 sta6-1 mt+	N - [HS -N]	t=4.0h
SRP038751	SRR1174406	SRS560424	Starch-null, Acetate-addition (Time Course)	CC-4348 sta6-1 mt+	N - [HS -N]	t=8.0h
SRP038751	SRR1174407	SRS560425	Starch-null, Acetate-addition (Time Course)	CC-4348 sta6-1 mt+	N - [HS -N]	t=12h
SRP038751	SRR1174408	SRS560426	Starch-null, Acetate-addition (Time Course)	CC-4348 sta6-1 mt+	N - [HS -N]	t=24h
SRP038751	SRR1174409	SRS560427	Starch-null, Acetate-addition (Time Course)	CC-4348 sta6-1 mt+	N - [HS -N]	t=48h
SRP038751	SRR1174410	SRS560428	Starch-null, Acetate-addition (Time Course)	CC-4348 sta6-1 mt+	N - [HS -N], Acetate + [20 mM]	t=0.0h
SRP038751	SRR1174411	SRS560429	Starch-null, Acetate-addition (Time Course)	CC-4348 sta6-1 mt+	N - [HS -N], Acetate + [20 mM]	t=0.5h
SRP038751	SRR1174412	SRS560430	Starch-null, Acetate-addition (Time Course)	CC-4348 sta6-1 mt+	N - [HS -N], Acetate + [20 mM]	t=2.0h
SRP038751	SRR1174413	SRS560431	Starch-null, Acetate-addition (Time Course)	CC-4348 sta6-1 mt+	N - [HS -N], Acetate + [20 mM]	t=4.0h
SRP038751	SRR1174414	SRS560400	Starch-null, Acetate-addition (Time Course)	CC-4348 sta6-1 mt+	N - [HS -N], Acetate + [20 mM]	t=8.0h
SRP038751	SRR1174415	SRS560399	Starch-null, Acetate-addition (Time Course)	CC-4348 sta6-1 mt+	N - [HS -N], Acetate + [20 mM]	t=12h
SRP038751	SRR1174417	SRS560432	Starch-null, Acetate-addition (Time Course)	CC-4348 sta6-1 mt+	N - [HS -N], Acetate + [20 mM]	t=48h
SRP031856	SRR1015638	SRS493181	Starch-null, N-deprivation (Time Course)	CC-4348 sta6-1 mt+	Standard	
SRP031856	SRR1015639	SRS493181	Starch-null, N-deprivation (Time Course)	CC-4348 sta6-1 mt+	Standard	
SRP031856	SRR1015640	SRS493182	Starch-null, N-deprivation (Time Course)	CC-4348 sta6-1 mt+	N - [TAP -N]	t=0.5h
SRP031856	SRR1015641	SRS493182	Starch-null, N-deprivation (Time Course)	CC-4348 sta6-1 mt+	N - [TAP -N]	t=0.5h

SRP031856	SRR1015642	SRS493183	Starch-null, N-deprivation (Time Course)	CC-4348 sta6-1 mt+	N - [TAP -N]	t=2.0h
SRP031856	SRR1015643	SRS493183	Starch-null, N-deprivation (Time Course)	CC-4348 sta6-1 mt+	N - [TAP -N]	t=2.0h
SRP031856	SRR1015644	SRS493184	Starch-null, N-deprivation (Time Course)	CC-4348 sta6-1 mt+	N - [TAP -N]	t=4.0h
SRP031856	SRR1015645	SRS493184	Starch-null, N-deprivation (Time Course)	CC-4348 sta6-1 mt+	N - [TAP -N]	t=4.0h
SRP031856	SRR1015646	SRS493185	Starch-null, N-deprivation (Time Course)	CC-4348 sta6-1 mt+	N - [TAP -N]	t=8.0h
SRP031856	SRR1015647	SRS493185	Starch-null, N-deprivation (Time Course)	CC-4348 sta6-1 mt+	N - [TAP -N]	t=8.0h
SRP031856	SRR1015648	SRS493186	Starch-null, N-deprivation (Time Course)	CC-4348 sta6-1 mt+	N - [TAP -N]	t=12h
SRP031856	SRR1015649	SRS493186	Starch-null, N-deprivation (Time Course)	CC-4348 sta6-1 mt+	N - [TAP -N]	t=12h
SRP031856	SRR1015650	SRS493187	Starch-null, N-deprivation (Time Course)	CC-4348 sta6-1 mt+	N - [TAP -N]	t=24h
SRP031856	SRR1015651	SRS493187	Starch-null, N-deprivation (Time Course)	CC-4348 sta6-1 mt+	N - [TAP -N]	t=24h
SRP031856	SRR1015652	SRS493188	Starch-null, N-deprivation (Time Course)	CC-4348 sta6-1 mt+	N - [TAP -N]	t=48h
SRP031856	SRR1015653	SRS493188	Starch-null, N-deprivation (Time Course)	CC-4348 sta6-1 mt+	N - [TAP -N]	t=48h
SRP031856	SRR1015654	SRS493189	Starch-null, N-deprivation (Time Course)	CC-4349 cw15 mt-	N - [TAP -N]	t=0.5h
SRP031856	SRR1015655	SRS493190	Starch-null, N-deprivation (Time Course)	CC-4349 cw15 mt-	N - [TAP -N]	t=4.0h
SRP031856	SRR1015656	SRS493191	Starch-null, N-deprivation (Time Course)	CC-4349 cw15 mt-	N - [TAP -N]	t=48h
SRP031856	SRR1015657	SRS493192	Starch-null, N-deprivation (Time Course)	CC-4348 sta6-1 mt+	N - [TAP -N]	t=0.5h
SRP031856	SRR1015658	SRS493193	Starch-null, N-deprivation (Time Course)	CC-4348 sta6-1 mt+	N - [TAP -N]	t=4.0h
SRP031856	SRR1015659	SRS493194	Starch-null, N-deprivation (Time Course)	CC-4348 sta6-1 mt+	N - [TAP -N]	t=48h
SRP031856	SRR1015660	SRS493195	Starch-null, N-deprivation (Time Course)	CC-4565 cw15 sta6-1::ARG7 STA6-complemented mt+	N - [TAP -N]	t=0.5h
SRP031856	SRR1015661	SRS493196	Starch-null, N-deprivation (Time Course)	CC-4565 cw15 sta6-1::ARG7 STA6-complemented mt+	N - [TAP -N]	t=4.0h
SRP031856	SRR1015662	SRS493197	Starch-null, N-deprivation (Time Course)	CC-4565 cw15 sta6-1::ARG7 STA6-complemented mt+	N - [TAP -N]	t=48h
SRP031856	SRR1015663	SRS493198	Starch-null, N-deprivation (Time Course)	CC-4566 cw15 sta6-1::ARG7 STA6-complemented mt+	N - [TAP -N]	t=0.5h
SRP031856	SRR1015664	SRS493199	Starch-null, N-deprivation (Time Course)	CC-4566 cw15 sta6-1::ARG7 STA6-complemented mt+	N - [TAP -N]	t=4.0h
SRP031856	SRR1015665	SRS493200	Starch-null, N-deprivation (Time Course)	CC-4566 cw15 sta6-1::ARG7 STA6-complemented mt+	N - [TAP -N]	t=48h
SRP031856	SRR1015666	SRS493201	Starch-null, N-deprivation (Time Course)	CC-4567 cw15 sta6-1::ARG7 STA6-complemented mt+	N - [TAP -N]	t=0.5h
SRP031856	SRR1015667	SRS493202	Starch-null, N-deprivation (Time Course)	CC-4567 cw15 sta6-1::ARG7 STA6-complemented mt+	N - [TAP -N]	t=4.0h
SRP031856	SRR1015668	SRS493203	Starch-null, N-deprivation (Time Course)	CC-4567 cw15 sta6-1::ARG7 STA6-complemented mt+	N - [TAP -N]	t=48h
SRP017044	SRR611223	SRS374130	crr1-mutant, O_2-deprivation (Time Course)	CC-124 wild type mt-	Standard	
SRP017044	SRR611224	SRS374131	crr1-mutant, O_2-deprivation (Time Course)	CC-124 wild type mt-	O₂ -, Dark +	t=0.5h
SRP017044	SRR611225	SRS374132	crr1-mutant, O_2-deprivation (Time Course)	CC-124 wild type mt-	O₂ -, Dark +	t=6.0h
SRP017044	SRR611238	SRS374139	crr1-mutant, O_2-deprivation (Time Course)	CC-3960 arg7 crr1-2::ble mt+	Standard	
SRP017044	SRR611239	SRS374139	crr1-mutant, O_2-deprivation (Time Course)	CC-3960 arg7 crr1-2::ble mt+	Standard	
SRP017044	SRR611240	SRS374140	crr1-mutant, O_2-deprivation (Time Course)	CC-3960 arg7 crr1-2::ble mt+	O₂ -, Dark +	t=0.5h
SRP017044	SRR611241	SRS374141	crr1-mutant, O_2-deprivation (Time Course)	CC-3960 arg7 crr1-2::ble mt+	O₂ -, Dark +	t=6.0h
SRP017044	SRR611242	SRS374141	crr1-mutant, O_2-deprivation (Time Course)	CC-3960 arg7 crr1-2::ble mt+	O₂ -, Dark +	t=6.0h
SRP017044	SRR611232	SRS374136	crr1-mutant, O_2-deprivation (Time Course)	CRR1-complemented from CC-3960 arg7 crr1-2::ble mt+	Standard	
SRP017044	SRR611233	SRS374136	crr1-mutant, O_2-deprivation (Time Course)	CRR1-complemented from CC-3960 arg7 crr1-2::ble mt+	Standard	
SRP017044	SRR611234	SRS374137	crr1-mutant, O_2-deprivation (Time Course)	CRR1-complemented from CC-3960 arg7 crr1-2::ble mt+	O₂ -, Dark +	t=0.5h

SRP017044	SRR611235	SRS374137	crr1-mutant, O_2-deprivation (Time Course)	CRR1-complemented from CC-3960 arg7 crr1-2::ble mt+	O₂ -, Dark +	t=0.5h
SRP017044	SRR611236	SRS374138	crr1-mutant, O_2-deprivation (Time Course)	CRR1-complemented from CC-3960 arg7 crr1-2::ble mt+	O₂ -, Dark +	t=6.0h
SRP017044	SRR611237	SRS374138	crr1-mutant, O_2-deprivation (Time Course)	CRR1-complemented from CC-3960 arg7 crr1-2::ble mt+	O₂ -, Dark +	t=6.0h
SRP017044	SRR611226	SRS374133	crr1-mutant, O_2-deprivation (Time Course)	CRR1dCys-complemented from CC-3960 arg7 crr1-2::ble mt+	Standard	
SRP017044	SRR611227	SRS374133	crr1-mutant, O_2-deprivation (Time Course)	CRR1dCys-complemented from CC-3960 arg7 crr1-2::ble mt+	Standard	
SRP017044	SRR611228	SRS374134	crr1-mutant, O_2-deprivation (Time Course)	CRR1dCys-complemented from CC-3960 arg7 crr1-2::ble mt+	O₂ -, Dark +	t=0.5h
SRP017044	SRR611229	SRS374134	crr1-mutant, O_2-deprivation (Time Course)	CRR1dCys-complemented from CC-3960 arg7 crr1-2::ble mt+	O₂ -, Dark +	t=0.5h
SRP017044	SRR611230	SRS374135	crr1-mutant, O_2-deprivation (Time Course)	CRR1dCys-complemented from CC-3960 arg7 crr1-2::ble mt+	O₂ -, Dark +	t=6.0h
SRP017044	SRR611231	SRS374135	crr1-mutant, O_2-deprivation (Time Course)	CRR1dCys-complemented from CC-3960 arg7 crr1-2::ble mt+	O₂ -, Dark +	t=6.0h
SRP018835	SRR764608	SRS397925	Fe-deprivation (Time Course)	CC-4532 Mets strain 2137 mt-	Standard	
SRP018835	SRR764609	SRS397925	Fe-deprivation (Time Course)	CC-4532 Mets strain 2137 mt-	Standard	
SRP018835	SRR764610	SRS397926	Fe-deprivation (Time Course)	CC-4532 Mets strain 2137 mt-	Fe - [0 mM]	t=0.5h
SRP018835	SRR764611	SRS397926	Fe-deprivation (Time Course)	CC-4532 Mets strain 2137 mt-	Fe - [0 mM]	t=0.5h
SRP018835	SRR764612	SRS397927	Fe-deprivation (Time Course)	CC-4532 Mets strain 2137 mt-	Fe - [0 mM]	t=1.0h
SRP018835	SRR764613	SRS397927	Fe-deprivation (Time Course)	CC-4532 Mets strain 2137 mt-	Fe - [0 mM]	t=1.0h
SRP018835	SRR764614	SRS397928	Fe-deprivation (Time Course)	CC-4532 Mets strain 2137 mt-	Fe - [0 mM]	t=2.0h
SRP018835	SRR764615	SRS397928	Fe-deprivation (Time Course)	CC-4532 Mets strain 2137 mt-	Fe - [0 mM]	t=2.0h
SRP018835	SRR764616	SRS397929	Fe-deprivation (Time Course)	CC-4532 Mets strain 2137 mt-	Fe - [0 mM]	t=4.0h
SRP018835	SRR764617	SRS397929	Fe-deprivation (Time Course)	CC-4532 Mets strain 2137 mt-	Fe - [0 mM]	t=4.0h
SRP018835	SRR764618	SRS397930	Fe-deprivation (Time Course)	CC-4532 Mets strain 2137 mt-	Fe - [0 mM]	t=8.0h
SRP018835	SRR764619	SRS397930	Fe-deprivation (Time Course)	CC-4532 Mets strain 2137 mt-	Fe - [0 mM]	t=8.0h
SRP018835	SRR764620	SRS397931	Fe-deprivation (Time Course)	CC-4532 Mets strain 2137 mt-	Fe - [0 mM]	t=12h
SRP018835	SRR764621	SRS397931	Fe-deprivation (Time Course)	CC-4532 Mets strain 2137 mt-	Fe - [0 mM]	t=12h
SRP018835	SRR764622	SRS397932	Fe-deprivation (Time Course)	CC-4532 Mets strain 2137 mt-	Fe - [0 mM]	t=24h
SRP018835	SRR764623	SRS397932	Fe-deprivation (Time Course)	CC-4532 Mets strain 2137 mt-	Fe - [0 mM]	t=24h
SRP018835	SRR764624	SRS397933	Fe-deprivation (Time Course)	CC-4532 Mets strain 2137 mt-	Fe - [0 mM]	t=48h
SRP018835	SRR764625	SRS397933	Fe-deprivation (Time Course)	CC-4532 Mets strain 2137 mt-	Fe - [0 mM]	t=48h
SRP014795	SRR537000	SRS355411	hmox1-mutant, Bilin-addition (Time Course)	CC-4051 4A+ mt+	Standard	
SRP014795	SRR537001	SRS355412	hmox1-mutant, Bilin-addition (Time Course)	CC-4051 4A+ mt+	Standard	
SRP014795	SRR537002	SRS355413	hmox1-mutant, Bilin-addition (Time Course)	CC-4051 4A+ mt+	Light +	t=0.5h
SRP014795	SRR537003	SRS355414	hmox1-mutant, Bilin-addition (Time Course)	CC-4051 4A+ mt+	Light +	t=0.5h
SRP014795	SRR537004	SRS355415	hmox1-mutant, Bilin-addition (Time Course)	CC-4051 4A+ mt+	Biliverdin Ixα + [0.1 mM]	
SRP014795	SRR537005	SRS355416	hmox1-mutant, Bilin-addition (Time Course)	CC-4051 4A+ mt+	Biliverdin Ixα + [0.1 mM]	
SRP014795	SRR537006	SRS355417	hmox1-mutant, Bilin-addition (Time Course)	CC-4051 4A+ mt+	Biliverdin Ixα + [0.1 mM], Light +	t=0.5h
SRP014795	SRR537007	SRS355418	hmox1-mutant, Bilin-addition (Time Course)	CC-4051 4A+ mt+	Biliverdin Ixα + [0.1 mM], Light +	t=0.5h
SRP014795	SRR537008	SRS355419	hmox1-mutant, Bilin-addition (Time Course)	hmox1-mutated from CC-4051 4A+ mt+	Standard	
SRP014795	SRR537009	SRS355420	hmox1-mutant, Bilin-addition (Time Course)	hmox1-mutated from CC-4051 4A+ mt+	Standard	
SRP014795	SRR537010	SRS355421	hmox1-mutant, Bilin-addition (Time Course)	hmox1-mutated from CC-4051 4A+ mt+	Light +	t=0.5h

SRP014795	SRR537011	SRS355422	hmox1-mutant, Bilin-addition (Time Course)	hmox1-mutated from CC-4051 4A+ mt+	Light +	t=0.5h
SRP014795	SRR537012	SRS355423	hmox1-mutant, Bilin-addition (Time Course)	hmox1-mutated from CC-4051 4A+ mt+	Biliverdin Ixα + [0.1 mM]	
SRP014795	SRR537013	SRS355424	hmox1-mutant, Bilin-addition (Time Course)	hmox1-mutated from CC-4051 4A+ mt+	Biliverdin Ixα + [0.1 mM]	
SRP014795	SRR537014	SRS355425	hmox1-mutant, Bilin-addition (Time Course)	hmox1-mutated from CC-4051 4A+ mt+	Biliverdin Ixα + [0.1 mM], Light +	t=0.5h
SRP014795	SRR537015	SRS355426	hmox1-mutant, Bilin-addition (Time Course)	hmox1-mutated from CC-4051 4A+ mt+	Biliverdin Ixα + [0.1 mM], Light +	t=0.5h
SRP010563	SRR402027	SRS290193	Fe-addition/deprivation	CC-1021 wild type mt+	Fe + [20 mM]	
SRP010563	SRR402028	SRS290194	Fe-addition/deprivation	CC-1021 wild type mt+	Fe + [20 mM]	
SRP010563	SRR402029	SRS290195	Fe-addition/deprivation	CC-1021 wild type mt+	Fe + [20 mM]	
SRP010563	SRR402030	SRS290196	Fe-addition/deprivation	CC-1021 wild type mt+	Fe + [20 mM]	
SRP010563	SRR402031	SRS290197	Fe-addition/deprivation	CC-1021 wild type mt+	Fe - [1 mM]	
SRP010563	SRR402032	SRS290198	Fe-addition/deprivation	CC-1021 wild type mt+	Fe - [1 mM]	
SRP010563	SRR402033	SRS290199	Fe-addition/deprivation	CC-1021 wild type mt+	Fe - [1 mM]	
SRP010563	SRR402034	SRS290200	Fe-addition/deprivation	CC-1021 wild type mt+	Fe - [1 mM]	
SRP010563	SRR402036	SRS290202	Fe-addition/deprivation	CC-1021 wild type mt+	Fe - [0.25 mM]	
SRP010563	SRR402037	SRS290203	Fe-addition/deprivation	CC-1021 wild type mt+	Fe - [0.25 mM]	
SRP010563	SRR402038	SRS290204	Fe-addition/deprivation	CC-1021 wild type mt+	Fe - [0.25 mM]	
SRP009466	SRR385610	SRS281061	cia5-mutant, CO_2-addition/deprivation	CC-125 wild type mt+	CO₂ + [50000 ppm]	
SRP009466	SRR385611	SRS281061	cia5-mutant, CO_2-addition/deprivation	CC-125 wild type mt+	CO₂ + [50000 ppm]	
SRP009466	SRR385608	SRS281060	cia5-mutant, CO_2-addition/deprivation	CC-125 wild type mt+	CO₂ - [300-500 ppm]	
SRP009466	SRR385609	SRS281060	cia5-mutant, CO_2-addition/deprivation	CC-125 wild type mt+	CO₂ - [300-500 ppm]	
SRP009466	SRR385613	SRS281062	cia5-mutant, CO_2-addition/deprivation	CC-125 wild type mt+	CO₂ - [100-200 ppm]	
SRP009466	SRR385616	SRS281064	cia5-mutant, CO_2-addition/deprivation	CC-2702 cia5	CO₂ + [50000 ppm]	
SRP009466	SRR385617	SRS281064	cia5-mutant, CO_2-addition/deprivation	CC-2702 cia5	CO₂ + [50000 ppm]	
SRP009466	SRR385614	SRS281063	cia5-mutant, CO_2-addition/deprivation	CC-2702 cia5	CO₂ - [300-500 ppm]	
SRP009466	SRR385615	SRS281063	cia5-mutant, CO_2-addition/deprivation	CC-2702 cia5	CO₂ - [300-500 ppm]	
SRP009466	SRR385618	SRS281065	cia5-mutant, CO_2-addition/deprivation	CC-2702 cia5	CO₂ - [100-200 ppm]	
SRP009466	SRR385619	SRS281065	cia5-mutant, CO_2-addition/deprivation	CC-2702 cia5	CO₂ - [100-200 ppm]	
SRP003630	SRR066643	SRS115154	N-addition/deprivation	CC-4619 cw15 nit1 mt+	N + [10 mM]	
SRP003630	SRR066644	SRS115155	N-addition/deprivation	CC-4619 cw15 nit1 mt+	N + [10 mM]	
SRP003630	SRR066645	SRS115156	N-addition/deprivation	CC-4619 cw15 nit1 mt+	N + [10 mM]	
SRP003630	SRR066646	SRS115157	N-addition/deprivation	CC-4619 cw15 nit1 mt+	N - [TAP -N]	
SRP003630	SRR066647	SRS115158	N-addition/deprivation	CC-4619 cw15 nit1 mt+	N - [TAP -N]	
SRP003630	SRR066648	SRS115159	N-addition/deprivation	CC-4619 cw15 nit1 mt+	N - [TAP -N]	
SRP010084	SRR394058	SRS284624	H_2O_2-addition (Time Course)	CC-1021 wild type mt+	H₂O₂ +	t=0.0h
SRP010084	SRR394059	SRS284624	H_2O_2-addition (Time Course)	CC-1021 wild type mt+	H₂O₂ +	t=0.0h
SRP010084	SRR394060	SRS284625	H_2O_2-addition (Time Course)	CC-1021 wild type mt+	H₂O₂ +	t=0.5h
SRP010084	SRR394061	SRS284625	H_2O_2-addition (Time Course)	CC-1021 wild type mt+	H₂O₂ +	t=0.5h
SRP010084	SRR394062	SRS284626	H_2O_2-addition (Time Course)	CC-1021 wild type mt+	H₂O₂ +	t=1.0h
SRP010084	SRR394063	SRS284626	H_2O_2-addition (Time Course)	CC-1021 wild type mt+	H₂O₂ +	t=1.0h
ERP001997	ERR202884	ERS091931	Development (Gametogenesis)	CC-1691 y1 mt-	Standard	
ERP001997	ERR202855	ERS091926	Development (Gametogenesis)	CC-1691 y1 mt-	Lys +	
ERP001997	ERR202879	ERS091926	Development (Gametogenesis)	CC-1691 y1 mt-	Lys +	
ERP001997	ERR202857	ERS091928	Development (Gametogenesis)	CC-1690 wild type mt+ / CC-1691 y1 mt-	Standard	Synchronised Vegetative Cells
ERP001997	ERR202873	ERS091944	Development (Gametogenesis)	CC-1690 wild type mt+ / CC-1691 y1 mt-	Standard	Unsynchronised Vegetative Cells

ERP001997	ERR411598	ERS005183	Development (Gametogenesis)	?	?	
SRP017616	SRR638709	SRS380944	Development (Deflagellation)	CC-125 wild type mt+	Standard	
SRP017616	SRR638710	SRS380945	Development (Deflagellation)	CC-125 wild type mt+	pH-shock + (Induce Ciliogenesis)	t=3min
SRP017616	SRR638711	SRS380946	Development (Deflagellation)	CC-125 wild type mt+	pH-shock + (Induce Ciliogenesis)	t=10min
SRP017616	SRR638712	SRS380947	Development (Deflagellation)	CC-125 wild type mt+	pH-shock + (Induce Ciliogenesis)	t=30min
SRP017616	SRR638713	SRS380948	Development (Deflagellation)	CC-125 wild type mt+	pH-shock + (Induce Ciliogenesis)	t=60min
SRP005483	SRR096519	SRS160728	crr1-mutant, Cu-addition/deprivation	CC-1021 wild type mt+	Cu +	
SRP005483	SRR096520	SRS160730	crr1-mutant, Cu-addition/deprivation	CC-1021 wild type mt+	Cu +	
SRP005483	SRR096517	SRS160724	crr1-mutant, Cu-addition/deprivation	CC-1021 wild type mt+	Cu -	
SRP005483	SRR096518	SRS160726	crr1-mutant, Cu-addition/deprivation	CC-1021 wild type mt+	Cu -	
SRP008996	SRR353961	SRS267333	gun4-mutant	cw15 mt- (Harris, 1989)	Standard	
SRP008996	SRR353969	SRS267335	gun4-mutant	cw15 mt- (Harris, 1989)	Standard	
SRP008996	SRR353965	SRS267334	gun4-mutant	gun4-mutated from cw15 mt- (Harris, 1989)	Standard	
SRP008996	SRR353973	SRS267336	gun4-mutant	gun4-mutated from cw15 mt- (Harris, 1989)	Standard	
SRP009273	SRR363979	SRS270135	sor1-mutant	sor1 4A+	Standard	
SRP010062	SRR393785	SRS284503	N-deprivation (Time Course)	CC-3269 wild type mt+	N - [TAP -N]	t=4.0h
