# Supplementary material for: ALCOdb: Gene Coexpression Database for Microalgae
Source: Plant Cell Physiol. 2015 Dec 7;57(1):e3. doi: 10.1093/pcp/pcv190 (PMC4722175; doi:10.1093/pcp/pcv190)
Supplement: Supplementary Data [file supp_pcv190_suppl_data.zip › pcp-2015-e-00476-File007.pdf]

**Supplementary Table S3: A summary of RNA-seq data sets used in DEG detection.**

| ID | Title | Summary | Strain1 | Condition1 | Data1 | Strain2 | Condition2 | Data2 |
|---|---|---|---|---|---|---|---|---|
| E001 | N depletion | Effect of N depletion | CC-4349 cw15 mt- | Standard | SRR1174385 | CC-4349 cw15 mt- | N - (30 minutes) | SRR1174386 |
| E002 | N depletion | Effect of N depletion | CC-4349 cw15 mt- | Standard | SRR1174385 | CC-4349 cw15 mt- | N - (2 hours) | SRR1174387 |
| E003 | N depletion | Effect of N depletion | CC-4349 cw15 mt- | Standard | SRR1174385 | CC-4349 cw15 mt- | N - (4 hours) | SRR1174388 |
| E004 | N depletion | Effect of N depletion | CC-4349 cw15 mt- | Standard | SRR1174385 | CC-4349 cw15 mt- | N - (8 hours) | SRR1174389 |
| E005 | N depletion | Effect of N depletion | CC-4349 cw15 mt- | Standard | SRR1174385 | CC-4349 cw15 mt- | N - (12 hours) | SRR1174390 |
| E006 | N depletion | Effect of N depletion | CC-4349 cw15 mt- | Standard | SRR1174385 | CC-4349 cw15 mt- | N - (24 hours) | SRR1174391 |
| E007 | N depletion | Effect of N depletion | CC-4349 cw15 mt- | Standard | SRR1174385 | CC-4349 cw15 mt- | N - (48 hours) | SRR1174392 |
| E008 | Acetate repletion | Effect of Acetate repletion | CC-4349 cw15 mt- | Standard | SRR1174393 | CC-4349 cw15 mt- | Acetate + (30 minutes) | SRR1174394 |
| E009 | Acetate repletion | Effect of Acetate repletion | CC-4349 cw15 mt- | Standard | SRR1174393 | CC-4349 cw15 mt- | Acetate + (2 hours) | SRR1174395 |
| E010 | Acetate repletion | Effect of Acetate repletion | CC-4349 cw15 mt- | Standard | SRR1174393 | CC-4349 cw15 mt- | Acetate + (4 hours) | SRR1174396 |
| E011 | Acetate repletion | Effect of Acetate repletion | CC-4349 cw15 mt- | Standard | SRR1174393 | CC-4349 cw15 mt- | Acetate + (8 hours) | SRR1174397 |
| E012 | Acetate repletion | Effect of Acetate repletion | CC-4349 cw15 mt- | Standard | SRR1174393 | CC-4349 cw15 mt- | Acetate + (12 hours) | SRR1174398 |
| E013 | Acetate repletion | Effect of Acetate repletion | CC-4349 cw15 mt- | Standard | SRR1174393 | CC-4349 cw15 mt- | Acetate + (24 hours) | SRR1174399 |
| E014 | Acetate repletion | Effect of Acetate repletion | CC-4349 cw15 mt- | Standard | SRR1174393 | CC-4349 cw15 mt- | Acetate + (48 hours) | SRR1174400 |
| E015 | N depletion | Effect of N depletion | CC-4348 sta6-1 mt+ | Standard | SRR1174402 | CC-4348 sta6-1 mt+ | N - (30 minutes) | SRR1174403 |
| E016 | N depletion | Effect of N depletion | CC-4348 sta6-1 mt+ | Standard | SRR1174402 | CC-4348 sta6-1 mt+ | N - (4 hours) | SRR1174405 |
| E017 | N depletion | Effect of N depletion | CC-4348 sta6-1 mt+ | Standard | SRR1174402 | CC-4348 sta6-1 mt+ | N - (8 hours) | SRR1174406 |
| E018 | N depletion | Effect of N depletion | CC-4348 sta6-1 mt+ | Standard | SRR1174402 | CC-4348 sta6-1 mt+ | N - (12 hours) | SRR1174407 |
| E019 | N depletion | Effect of N depletion | CC-4348 sta6-1 mt+ | Standard | SRR1174402 | CC-4348 sta6-1 mt+ | N - (24 hours) | SRR1174408 |
| E020 | N depletion | Effect of N depletion | CC-4348 sta6-1 mt+ | Standard | SRR1174402 | CC-4348 sta6-1 mt+ | N - (48 hours) | SRR1174409 |
| E021 | Acetate repletion | Effect of Acetate repletion | CC-4348 sta6-1 mt+ | Standard | SRR1174410 | CC-4348 sta6-1 mt+ | Acetate + (30 minutes) | SRR1174411 |
| E022 | Acetate repletion | Effect of Acetate repletion | CC-4348 sta6-1 mt+ | Standard | SRR1174410 | CC-4348 sta6-1 mt+ | Acetate + (2 hours) | SRR1174412 |
| E023 | Acetate repletion | Effect of Acetate repletion | CC-4348 sta6-1 mt+ | Standard | SRR1174410 | CC-4348 sta6-1 mt+ | Acetate + (4 hours) | SRR1174413 |
| E024 | Acetate repletion | Effect of Acetate repletion | CC-4348 sta6-1 mt+ | Standard | SRR1174410 | CC-4348 sta6-1 mt+ | Acetate + (8 hours) | SRR1174414 |
| E025 | Acetate repletion | Effect of Acetate repletion | CC-4348 sta6-1 mt+ | Standard | SRR1174410 | CC-4348 sta6-1 mt+ | Acetate + (12 hours) | SRR1174415 |
| E026 | Acetate repletion | Effect of Acetate repletion | CC-4348 sta6-1 mt+ | Standard | SRR1174410 | CC-4348 sta6-1 mt+ | Acetate + (48 hours) | SRR1174417 |
| E027 | N depletion | Effect of N depletion | CC-4348 sta6-1 mt+ | Standard | SRR1015638,SRR1015639 | CC-4348 sta6-1 mt+ | N - (30 minutes) | SRR1015640,SRR1015641 |
| E028 | N depletion | Effect of N depletion | CC-4348 sta6-1 mt+ | Standard | SRR1015638,SRR1015639 | CC-4348 sta6-1 mt+ | N - (2 hours) | SRR1015642,SRR1015643 |
| E029 | N depletion | Effect of N depletion | CC-4348 sta6-1 mt+ | Standard | SRR1015638,SRR1015639 | CC-4348 sta6-1 mt+ | N - (4 hours) | SRR1015644,SRR1015645 |
| E030 | N depletion | Effect of N depletion | CC-4348 sta6-1 mt+ | Standard | SRR1015638,SRR1015639 | CC-4348 sta6-1 mt+ | N - (8 hours) | SRR1015646,SRR1015647 |
| E031 | N depletion | Effect of N depletion | CC-4348 sta6-1 mt+ | Standard | SRR1015638,SRR1015639 | CC-4348 sta6-1 mt+ | N - (12 hours) | SRR1015648,SRR1015649 |
| E032 | N depletion | Effect of N depletion | CC-4348 sta6-1 mt+ | Standard | SRR1015638,SRR1015639 | CC-4348 sta6-1 mt+ | N - (24 hours) | SRR1015650,SRR1015651 |
| E033 | N depletion | Effect of N depletion | CC-4348 sta6-1 mt+ | Standard | SRR1015638,SRR1015639 | CC-4348 sta6-1 mt+ | N - (48 hours) | SRR1015652,SRR1015653 |
| E034 | O2 depletion | Effect of O2 depletion | CC-124 wild type mt- | Standard | SRR611223 | CC-124 wild type mt- | Dark, Anoxic (30 minutes) | SRR611224 |
| E035 | O2 depletion | Effect of O2 depletion | CC-124 wild type mt- | Standard | SRR611223 | CC-124 wild type mt- | Dark, Anoxic (6 hours) | SRR611225 |
| E036 | O2 depletion | Effect of O2 depletion | CC-3960 arg7 crr1-2::ble mt+ | Standard | SRR611238,SRR611239 | CC-3960 arg7 crr1-2::ble mt+ | Dark, Anoxic (30 minutes) | SRR611240 |
| E037 | O2 depletion | Effect of O2 depletion | CC-3960 arg7 crr1-2::ble mt+ | Standard | SRR611238,SRR611239 | CC-3960 arg7 crr1-2::ble mt+ | Dark, Anoxic (6 hours) | SRR611241,SRR611242 |
| E038 | O2 depletion | Effect of O2 depletion | CRR1-complemented from CC-3960 arg7 crr1-2::ble mt+ | Standard | SRR611232,SRR611233 | CRR1-complemented from CC-3960 arg7 crr1-2::ble mt+ | Dark, Anoxic (30 minutes) | SRR611234,SRR611235 |
| E039 | O2 depletion | Effect of O2 depletion | CRR1-complemented from CC-3960 arg7 crr1-2::ble mt+ | Standard | SRR611232,SRR611233 | CRR1-complemented from CC-3960 arg7 crr1-2::ble mt+ | Dark, Anoxic (6 hours) | SRR611236,SRR611237 |
| E040 | O2 depletion | Effect of O2 depletion | CRR1dCys-complemented from CC-3960 arg7 crr1-2::ble mt+ | Standard | SRR611226,SRR611227 | CRR1dCys-complemented from CC-3960 arg7 crr1-2::ble mt+ | Dark, Anoxic (30 minutes) | SRR611228,SRR611229 |
| E041 | O2 depletion | Effect of O2 depletion | CRR1dCys-complemented from CC-3960 arg7 crr1-2::ble mt+ | Standard | SRR611226,SRR611227 | CRR1dCys-complemented from CC-3960 arg7 crr1-2::ble mt+ | Dark, Anoxic (6 hours) | SRR611230,SRR611231 |
| E042 | Fe depletion | Effect of Fe depletion | CC-4532 Mets strain 2137 mt- | Standard | SRR764608,SRR764609 | CC-4532 Mets strain 2137 mt- | Fe - (30 minutes) | SRR764610,SRR764611 |
| E043 | Fe depletion | Effect of Fe depletion | CC-4532 Mets strain 2137 mt- | Standard | SRR764608,SRR764609 | CC-4532 Mets strain 2137 mt- | Fe - (1 hour) | SRR764612,SRR764613 |
| E044 | Fe depletion | Effect of Fe depletion | CC-4532 Mets strain 2137 mt- | Standard | SRR764608,SRR764609 | CC-4532 Mets strain 2137 mt- | Fe - (2 hours) | SRR764614,SRR764615 |
| E045 | Fe depletion | Effect of Fe depletion | CC-4532 Mets strain 2137 mt- | Standard | SRR764608,SRR764609 | CC-4532 Mets strain 2137 mt- | Fe - (4 hours) | SRR764616,SRR764617 |
| E046 | Fe depletion | Effect of Fe depletion | CC-4532 Mets strain 2137 mt- | Standard | SRR764608,SRR764609 | CC-4532 Mets strain 2137 mt- | Fe - (8 hours) | SRR764618,SRR764619 |
| E047 | Fe depletion | Effect of Fe depletion | CC-4532 Mets strain 2137 mt- | Standard | SRR764608,SRR764609 | CC-4532 Mets strain 2137 mt- | Fe - (12 hours) | SRR764620,SRR764621 |
| E048 | Fe depletion | Effect of Fe depletion | CC-4532 Mets strain 2137 mt- | Standard | SRR764608,SRR764609 | CC-4532 Mets strain 2137 mt- | Fe - (24 hours) | SRR764622,SRR764623 |
| E049 | Fe depletion | Effect of Fe depletion | CC-4532 Mets strain 2137 mt- | Standard | SRR764608,SRR764609 | CC-4532 Mets strain 2137 mt- | Fe - (48 hours) | SRR764624,SRR764625 |
| E050 | Dark-to-Light transition | Effect of Dark-to-Light transition | CC-4051 4A+ mt+ | Standard | SRR537000,SRR537001 | CC-4051 4A+ mt+ | Light (30 minutes) | SRR537002,SRR537003 |
| E051 | Bilin repletion | Effect of Bilin repletion | CC-4051 4A+ mt+ | Standard | SRR537000,SRR537001 | CC-4051 4A+ mt+ | Biliverdin + | SRR537004,SRR537005 |
| E052 | Dark-to-Light transition | Effect of Dark-to-Light transition | CC-4051 4A+ mt+ | Biliverdin + | SRR537004,SRR537005 | CC-4051 4A+ mt+ | Biliverdin +, Light (30 minutes) | SRR537006,SRR537007 |
| E053 | Bilin repletion | Effect of Bilin repletion on Dark-to-Light transition | CC-4051 4A+ mt+ | Light (30 minutes) | SRR537002,SRR537003 | CC-4051 4A+ mt+ | Biliverdin +, Light (30 minutes) | SRR537006,SRR537007 |
| E054 | Dark-to-Light transition | Effect of Dark-to-Light transition | hmox1-mutated from CC-4051 4A+ mt+ | Standard | SRR537008,SRR537009 | hmox1-mutated from CC-4051 4A+ mt+ | Light (30 minutes) | SRR537010,SRR537011 |
| E055 | Bilin repletion | Effect of Bilin repletion | hmox1-mutated from CC-4051 4A+ mt+ | Standard | SRR537008,SRR537009 | hmox1-mutated from CC-4051 4A+ mt+ | Biliverdin + (30 minutes) | SRR537012,SRR537013 |
| E056 | Dark-to-Light transition | Effect of Dark-to-Light transition | hmox1-mutated from CC-4051 4A+ mt+ | Biliverdin + (30 minutes) | SRR537012,SRR537013 | hmox1-mutated from CC-4051 4A+ mt+ | Light, Biliverdin + (30 minutes) | SRR537014,SRR537015 |
| E057 | Bilin repletion | Effect of Bilin repletion on Dark-to-Light transition | hmox1-mutated from CC-4051 4A+ mt+ | Light (30 minutes) | SRR537010,SRR537011 | hmox1-mutated from CC-4051 4A+ mt+ | Light, Biliverdin + (30 minutes) | SRR537014,SRR537015 |
| E058 | Fe depletion | Effect of Fe depletion | CC-1021 wild type mt+ | Fe [20 mM] | SRR402027,SRR402028,SRR402029,SRR402030 | CC-1021 wild type mt+ | Fe [1 mM] | SRR402031,SRR402032,SRR402033,SRR402034 |
| E059 | Fe depletion | Effect of Fe depletion | CC-1021 wild type mt+ | Fe [20 mM] | SRR402027,SRR402028,SRR402029,SRR402030 | CC-1021 wild type mt+ | Fe [0.25 mM] | SRR402036,SRR402037,SRR402038 |
| E060 | Fe depletion | Effect of Fe depletion | CC-1021 wild type mt+ | Fe [1 mM] | SRR402031,SRR402032,SRR402033,SRR402034 | CC-1021 wild type mt+ | Fe [0.25 mM] | SRR402036,SRR402037,SRR402038 |
| E061 | CO2 repletion | Effect of CO2 repletion | CC-125 wild type mt+ | CO2 [300-500 ppm] | SRR385608,SRR385609 | CC-125 wild type mt+ | CO2 [50000 ppm] | SRR385610,SRR385611 |
| E062 | CO2 depletion | Effect of CO2 depletion | CC-125 wild type mt+ | CO2 [300-500 ppm] | SRR385608,SRR385609 | CC-125 wild type mt+ | CO2 [100-200 ppm] | SRR385613 |
| E063 | CO2 repletion | Effect of CO2 repletion | CC-2702 cia5 | CO2 [300-500 ppm] | SRR385614,SRR385615 | CC-2702 cia5 | CO2 [50000 ppm] | SRR385616,SRR385617 |
| E064 | CO2 depletion | Effect of CO2 depletion | CC-2702 cia5 | CO2 [300-500 ppm] | SRR385614,SRR385615 | CC-2702 cia5 | CO2 [100-200 ppm] | SRR385618,SRR385619 |
| E065 | N depletion | Effect of N depletion | CC-4619 cw15 nit1 mt+ | Standard | SRR066643,SRR066644,SRR066645 | CC-4619 cw15 nit1 mt+ | N - (48 hours) | SRR066646,SRR066647,SRR066648 |
| E066 | H2O2 repletion | Effect of H2O2 repletion | CC-1021 wild type mt+ | Standard | SRR394058,SRR394059 | CC-1021 wild type mt+ | H2O2 + (30 minutes) | SRR394060,SRR394061 |
| E067 | H2O2 repletion | Effect of H2O2 repletion | CC-1021 wild type mt+ | Standard | SRR394058,SRR394059 | CC-1021 wild type mt+ | H2O2 + (1 hour) | SRR394062,SRR394063 |
| E068 | Low pH shock | Effect of pH shock (pH 4.0, 45 seconds) | CC-125 wild type mt+ | Standard | SRR638709 | CC-125 wild type mt+ | 3 minutes after pH shock | SRR638710 |
| E069 | Low pH shock | Effect of pH shock (pH 4.0, 45 seconds) | CC-125 wild type mt+ | Standard | SRR638709 | CC-125 wild type mt+ | 10 minutes after pH shock | SRR638711 |
| E070 | Low pH shock | Effect of pH shock (pH 4.0, 45 seconds) | CC-125 wild type mt+ | Standard | SRR638709 | CC-125 wild type mt+ | 30 minutes after pH shock | SRR638712 |
| E071 | Low pH shock | Effect of pH shock (pH 4.0, 45 seconds) | CC-125 wild type mt+ | Standard | SRR638709 | CC-125 wild type mt+ | 1 hour after pH shock | SRR638713 |
| E072 | Cu depletion | Effect of Cu depletion | CC-1021 wild type mt+ | Standard | SRR096519,SRR096520 | CC-1021 wild type mt+ | Cu - | SRR096517,SRR096518 |
| G001 | sta6 mutation | Effect of sta6 mutation | CC-4349 cw15 mt- | Standard | SRR1174384 | CC-4348 sta6-1 mt+ | Standard | SRR1174401 |
| G002 | sta6 mutation | Effect of sta6 mutation | CC-4349 cw15 mt- | Standard | SRR1174385 | CC-4348 sta6-1 mt+ | Standard | SRR1174402 |
| G003 | sta6 mutation | Effect of sta6 mutation on N depletion | CC-4349 cw15 mt- | N - (30 minutes) | SRR1174386 | CC-4348 sta6-1 mt+ | N - (30 minutes) | SRR1174403 |
| G004 | sta6 mutation | Effect of sta6 mutation on N depletion | CC-4349 cw15 mt- | N - (4 hours) | SRR1174388 | CC-4348 sta6-1 mt+ | N - (4 hours) | SRR1174405 |
| G005 | sta6 mutation | Effect of sta6 mutation on N depletion | CC-4349 cw15 mt- | N - (8 hours) | SRR1174389 | CC-4348 sta6-1 mt+ | N - (8 hours) | SRR1174406 |
| G006 | sta6 mutation | Effect of sta6 mutation on N depletion | CC-4349 cw15 mt- | N - (12 hours) | SRR1174390 | CC-4348 sta6-1 mt+ | N - (12 hours) | SRR1174407 |

| | | | | | | | | |
|---|---|---|---|---|---|---|---|---|
| G007 | sta6 mutation | Effect of sta6 mutation on N depletion | CC-4349 cw15 mt- | N - (24 hours) | SRR1174391 | CC-4348 sta6-1 mt+ | N - (24 hours) | SRR1174408 |
| G008 | sta6 mutation | Effect of sta6 mutation on N depletion | CC-4349 cw15 mt- | N - (48 hours) | SRR1174392 | CC-4348 sta6-1 mt+ | N - (48 hours) | SRR1174409 |
| G009 | sta6 mutation | Effect of sta6 mutation | CC-4349 cw15 mt- | Standard | SRR1174393 | CC-4348 sta6-1 mt+ | Standard | SRR1174410 |
| G010 | sta6 mutation | Effect of sta6 mutation onAcetate repletion | CC-4349 cw15 mt- | Acetate + (30 minutes) | SRR1174394 | CC-4348 sta6-1 mt+ | Acetate + (30 minutes) | SRR1174411 |
| G011 | sta6 mutation | Effect of sta6 mutation onAcetate repletion | CC-4349 cw15 mt- | Acetate + (2 hours) | SRR1174395 | CC-4348 sta6-1 mt+ | Acetate + (2 hours) | SRR1174412 |
| G012 | sta6 mutation | Effect of sta6 mutation onAcetate repletion | CC-4349 cw15 mt- | Acetate + (4 hours) | SRR1174396 | CC-4348 sta6-1 mt+ | Acetate + (4 hours) | SRR1174413 |
| G013 | sta6 mutation | Effect of sta6 mutation onAcetate repletion | CC-4349 cw15 mt- | Acetate + (8 hours) | SRR1174397 | CC-4348 sta6-1 mt+ | Acetate + (8 hours) | SRR1174414 |
| G014 | sta6 mutation | Effect of sta6 mutation onAcetate repletion | CC-4349 cw15 mt- | Acetate + (12 hours) | SRR1174398 | CC-4348 sta6-1 mt+ | Acetate + (12 hours) | SRR1174415 |
| G015 | sta6 mutation | Effect of sta6 mutation onAcetate repletion | CC-4349 cw15 mt- | Acetate + (48 hours) | SRR1174400 | CC-4348 sta6-1 mt+ | Acetate + (48 hours) | SRR1174417 |
| G016 | sta6 mutation | Effect of sta6 mutation on N depletion | CC-4349 cw15 mt- | N - (30 minutes) | SRR1015654 | CC-4348 sta6-1 mt+ | N - (30 minutes) | SRR1015657 |
| G017 | sta6 mutation | Effect of sta6 mutation on N depletion | CC-4349 cw15 mt- | N - (4 hours) | SRR1015655 | CC-4348 sta6-1 mt+ | N - (4 hours) | SRR1015658 |
| G018 | sta6 mutation | Effect of sta6 mutation on N depletion | CC-4349 cw15 mt- | N - (48 hours) | SRR1015656 | CC-4348 sta6-1 mt+ | N - (48 hours) | SRR1015659 |
| G019 | sta6 complementation | Effect of sta6 complementation on N depletion | CC-4348 sta6-1 mt+ | N - (30 minutes) | SRR1015657 | CC-4565 cw15 sta6-1::ARG7 STA6-complemented mt+ | N - (30 minutes) | SRR1015660 |
| G020 | sta6 complementation | Effect of sta6 complementation on N depletion | CC-4348 sta6-1 mt+ | N - (4 hours) | SRR1015658 | CC-4565 cw15 sta6-1::ARG7 STA6-complemented mt+ | N - (4 hours) | SRR1015661 |
| G021 | sta6 complementation | Effect of sta6 complementation on N depletion | CC-4348 sta6-1 mt+ | N - (48 hours) | SRR1015659 | CC-4565 cw15 sta6-1::ARG7 STA6-complemented mt+ | N - (48 hours) | SRR1015662 |
| G022 | sta6 complementation | Effect of sta6 complementation on N depletion | CC-4348 sta6-1 mt+ | N - (30 minutes) | SRR1015657 | CC-4565 cw15 sta6-1::ARG7 STA6-complemented mt+ | N - (30 minutes) | SRR1015663 |
| G023 | sta6 complementation | Effect of sta6 complementation on N depletion | CC-4348 sta6-1 mt+ | N - (4 hours) | SRR1015658 | CC-4565 cw15 sta6-1::ARG7 STA6-complemented mt+ | N - (4 hours) | SRR1015664 |
| G024 | sta6 complementation | Effect of sta6 complementation on N depletion | CC-4348 sta6-1 mt+ | N - (48 hours) | SRR1015659 | CC-4565 cw15 sta6-1::ARG7 STA6-complemented mt+ | N - (48 hours) | SRR1015665 |
| G025 | sta6 complementation | Effect of sta6 complementation on N depletion | CC-4348 sta6-1 mt+ | N - (30 minutes) | SRR1015657 | CC-4565 cw15 sta6-1::ARG7 STA6-complemented mt+ | N - (30 minutes) | SRR1015666 |
| G026 | sta6 complementation | Effect of sta6 complementation on N depletion | CC-4348 sta6-1 mt+ | N - (4 hours) | SRR1015658 | CC-4565 cw15 sta6-1::ARG7 STA6-complemented mt+ | N - (4 hours) | SRR1015667 |
| G027 | sta6 complementation | Effect of sta6 complementation on N depletion | CC-4348 sta6-1 mt+ | N - (48 hours) | SRR1015659 | CC-4565 cw15 sta6-1::ARG7 STA6-complemented mt+ | N - (48 hours) | SRR1015668 |
| G028 | crr1 mutation | Effect of crr1 mutation | CC-124 wild type mt- | Standard | SRR611223 | CC-3960 arg7 crr1-2::ble mt+ | Standard | SRR611238,SRR611239 |
| G029 | crr1 mutation | Effect of crr1 mutation on O2 depletion | CC-124 wild type mt- | Dark, Anoxic (30 minutes) | SRR611224 | CC-3960 arg7 crr1-2::ble mt+ | Dark, Anoxic (30 minutes) | SRR611240 |
| G030 | crr1 mutation | Effect of crr1 mutation on O2 depletion | CC-124 wild type mt- | Dark, Anoxic (6 hours) | SRR611225 | CC-3960 arg7 crr1-2::ble mt+ | Dark, Anoxic (6 hours) | SRR611241,SRR611242 |
| G031 | crr1 complementation | Effect of crr1 complementation | CC-3960 arg7 crr1-2::ble mt+ | Standard | SRR611238,SRR611239 | CRR1-complemented from CC-3960 arg7 crr1-2::ble mt+ | Standard | SRR611232,SRR611233 |
| G032 | crr1 complementation | Effect of crr1 complementation on O2 depletion | CC-3960 arg7 crr1-2::ble mt+ | Dark, Anoxic (30 minutes) | SRR611240 | CRR1-complemented from CC-3960 arg7 crr1-2::ble mt+ | Dark, Anoxic (30 minutes) | SRR611234,SRR611235 |
| G033 | crr1 complementation | Effect of crr1 complementation on O2 depletion | CC-3960 arg7 crr1-2::ble mt+ | Dark, Anoxic (6 hours) | SRR611241,SRR611242 | CRR1-complemented from CC-3960 arg7 crr1-2::ble mt+ | Dark, Anoxic (6 hours) | SRR611236,SRR611237 |
| G034 | crr1 complementation | Effect of crr1-variant complementation | CC-3960 arg7 crr1-2::ble mt+ | Standard | SRR611238,SRR611239 | CRR1dCys-complemented from CC-3960 arg7 crr1-2::ble mt+ | Standard | SRR611226,SRR611227 |
| G035 | crr1 complementation | Effect of crr1-variant complementation on O2 depletion | CC-3960 arg7 crr1-2::ble mt+ | Dark, Anoxic (30 minutes) | SRR611240 | CRR1dCys-complemented from CC-3960 arg7 crr1-2::ble mt+ | Dark, Anoxic (30 minutes) | SRR611228,SRR611229 |
| G036 | crr1 complementation | Effect of crr1-variant complementation on O2 depletion | CC-3960 arg7 crr1-2::ble mt+ | Dark, Anoxic (6 hours) | SRR611241,SRR611242 | CRR1dCys-complemented from CC-3960 arg7 crr1-2::ble mt+ | Dark, Anoxic (6 hours) | SRR611230,SRR611231 |
| G037 | crr1 complementation | Effect of crr1-variant complementation | CRR1-complemented from CC-3960 arg7 crr1-2::ble mt+ | Standard | SRR611232,SRR611233 | CRR1dCys-complemented from CC-3960 arg7 crr1-2::ble mt+ | Standard | SRR611226,SRR611227 |
| G038 | crr1 complementation | Effect of crr1-variant complementation on O2 depletion | CRR1-complemented from CC-3960 arg7 crr1-2::ble mt+ | Dark, Anoxic (30 minutes) | SRR611234,SRR611235 | CRR1dCys-complemented from CC-3960 arg7 crr1-2::ble mt+ | Dark, Anoxic (30 minutes) | SRR611228,SRR611229 |
| G039 | crr1 complementation | Effect of crr1-variant complementation on O2 depletion | CRR1-complemented from CC-3960 arg7 crr1-2::ble mt+ | Dark, Anoxic (6 hours) | SRR611236,SRR611237 | CRR1dCys-complemented from CC-3960 arg7 crr1-2::ble mt+ | Dark, Anoxic (6 hours) | SRR611230,SRR611231 |
| G040 | hmox1 mutation | Effect of hmox1 mutationn | CC-4051 4A+ mt+ | Standard | SRR537000,SRR537001 | hmox1-mutated from CC-4051 4A+ mt+ | Standard | SRR537008,SRR537009 |
| G041 | hmox1 mutation | Effect of hmox1 mutationn on Dark-to-Light transition | CC-4051 4A+ mt+ | Light (30 minutes) | SRR537002,SRR537003 | hmox1-mutated from CC-4051 4A+ mt+ | Light (30 minutes) | SRR537010,SRR537011 |
| G042 | hmox1 mutation | Effect of hmox1 mutationn on Dark-to-Light transition | CC-4051 4A+ mt+ | Biliverdin + | SRR537004,SRR537005 | hmox1-mutated from CC-4051 4A+ mt+ | Biliverdin + | SRR537012,SRR537013 |
| G043 | hmox1 mutation | Effect of hmox1 mutationn on Dark-to-Light transition | CC-4051 4A+ mt+ | Biliverdin +, Light (30 minutes) | SRR537006,SRR537007 | hmox1-mutated from CC-4051 4A+ mt+ | Biliverdin +, Light (30 minutes) | SRR537014,SRR537015 |
| G044 | cia5 mutation | Effect of cia5 mutation | CC-125 wild type mt+ | CO2 [300-500 ppm] | SRR385608,SRR385609 | CC-2702 cia5 | CO2 [300-500 ppm] | SRR385614,SRR385615 |
| G045 | cia5 mutation | Effect of cia5 mutation on CO2 repletion | CC-125 wild type mt+ | CO2 [50000 ppm] | SRR385610,SRR385611 | CC-2702 cia5 | CO2 [50000 ppm] | SRR385616,SRR385617 |
| G046 | cia5 mutation | Effect of cia5 mutation on CO2 depletion | CC-125 wild type mt+ | CO2 [100-200 ppm] | SRR385613 | CC-2702 cia5 | CO2 [100-200 ppm] | SRR385618,SRR385619 |
| G047 | gun4 mutation | Effect of gun4 mutation | cw15 mt- (Harris, 1989) | Standard | SRR353961,SRR353969 | gun4-mutated from cw15 mt- (Harris, 1989) | Standard | SRR353965,SRR353973 |
